# Dairy-Inspired Coatings for Bone Implants from Whey Protein Isolate-Derived Self-Assembled Fibrils

**DOI:** 10.3390/ijms21155544

**Published:** 2020-08-03

**Authors:** Rebecca Rabe, Ute Hempel, Laurine Martocq, Julia K. Keppler, Jenny Aveyard, Timothy E. L. Douglas

**Affiliations:** 1 Division of Food Technology, Kiel University, 24118 Kiel, Germany; rebecca.rabe@gmx.com (R.R.); julia.keppler@wur.nl (J.K.K.); 2 Institute of Physiological Chemistry, Technische Universität Dresden, 01069 Dresden, Germany; hempel-u@mail.zih.tu-dresden.de; 3 Engineering Department, Lancaster University, Lancaster LA1 4YW, UK; t.douglas@lancaster.ac.uk; 4 Laboratory of Food Process Engineering, Wageningen University & Research AFSG, 6708 PB Wageningen, The Netherlands; 5 School of Engineering, University of Liverpool, Liverpool L69 3BX, UK; zippy78@liverpool.ac.uk; 6 Materials Science Institute (MSI), Lancaster University, Lancaster LA1 4YW, UK

**Keywords:** coating, stem cell, whey protein isolate, bone, fibril

## Abstract

To improve the integration of a biomaterial with surrounding tissue, its surface properties may be modified by adsorption of biomacromolecules, e.g., fibrils. Whey protein isolate (WPI), a dairy industry by-product, supports osteoblastic cell growth. WPI’s main component, β-lactoglobulin, forms fibrils in acidic solutions. In this study, aiming to develop coatings for biomaterials for bone contact, substrates were coated with WPI fibrils obtained at pH 2 or 3.5. Importantly, WPI fibrils coatings withstood autoclave sterilization and appeared to promote spreading and differentiation of human bone marrow stromal cells (hBMSC). In the future, WPI fibrils coatings could facilitate immobilization of biomolecules with growth stimulating or antimicrobial properties.

## 1. Introduction

Whey protein isolate (WPI) is a dairy industry by-product which contains > 95% protein, of which 75% is β-lactoglobulin [1]. Previous studies showed that WPI enhances cell proliferation and osteogenic differentiation and displays antibacterial properties [1,2,3]. Upon heating for several hours under acidic conditions (<pH 3), β-lactoglobulin degrades into smaller peptides, which undergo self-assembly to form amyloid fibrils several micrometers long and a few nm thick [4]. Heating at pH 3.5 leads to worm-like structures which consist of whole protein instead of peptides [5,6,7].

Better cell-biomaterial interactions and biomaterial integration into host tissue can be achieved by improving surface properties, e.g., by coatings. Fibrillar coatings have advantages including high surface/volume ratio, promoting fibril adhesion to substrates. Biologically active molecules can be immobilized on fibrils [8,9], which can form aligned superstructure scaffolds [10], improve cell line attachment and act as biomimetic cell culture platforms [11,12,13].

A commonly used fibrillar molecule used as implant coating materials is collagen, which is known to promote cell adhesion, spreading and proliferation [14,15,16,17,18]. Fibronectin is another commonly used molecule to improve cell adhesion, also in fibrillar form [19,20]. One advantage of WPI is its low cost, as it is a by-product of the dairy industry.

With the intention of developing coatings for biomaterials for bone contact, WPI fibrillar coatings were formed to support and enhance the spreading, attachment and differentiation of human bone marrow stromal cells (hBMSC), which have greater clinical relevance than cell lines. WPI fibrils were hypothesized to withstand autoclaving as WPI hydrogels do [21]. Autoclaving was preferred due to its ubiquity, clinical acceptance and low cost.

WPI fibrils were formed in solutions at two different pH values, 2 and 3.5. A WPI solution concentration of 2.5 wt% was used. WPI concentrations between 2.5 wt% and 5 wt% were found to result in a high fibril yield [3,4], and especially 2.5 wt% WPI is often used in WPI or β-Lactoglobulin fibril studies, due to the lower sample viscosity, which improves handling. Because the protein concentration also affects the aggregation kinetics and the morphology of the resulting aggregates [22,23,24], deviations from these ideal values can also reduce the comparability to other studies, and then be adsorbed onto substrates and imaged by scanning electron microscopy (SEM). Finally, autoclaved coatings were characterized using hBMSC.

## 2. Results and Discussion

**Figure 1:**
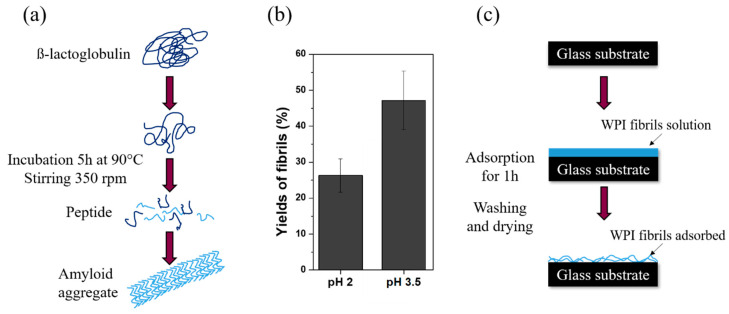
(**a**) Process of fibrils formation at pH 2 in solution; at pH 2, β-lactoglobulin denatures and hydrolyses at 90 °C. Specific peptides self-associate into the amyloid aggregates, which can consist of approximately three intertwined protofibrils. At pH 3.5, acid hydrolysis is reduced; therefore, non-hydrolyzed β-lactoglobulin assembles into worm-like aggregates, which are not amyloid but amyloid-like, and of different shape and morphology. (**b**) Fibrillar yield in solutions of different pH and (**c**) adsorption of whey protein isolate (WPI) fibrils on glass substrates.

WPI fibril formation (shown schematically in Figure 1a) was influenced by pH, as measurements of fibrillar yield at pH 2 (approximately 25%) and pH 3.5 (>40%) demonstrated (Figure 1b). Similar observations were reported previously [5,6,7]. Differences in yield are attributed to differences in the fibril building blocks, which are specific acid hydrolyzed peptides at pH 2 [4], but unspecific non-hydrolyzed proteins at pH 3.5 [5].

**Figure 2:**
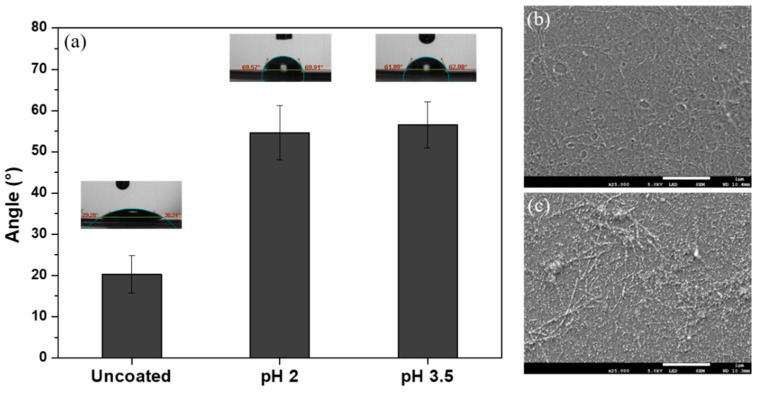
(**a**) Contact angle (CA) measurements of uncoated and fibrillar coated samples with solution at pH 2 and pH 3.5 and SEM images of fibrillar coatings obtained at (**b**) pH 2 and (**c**) pH 3.5 (scale bar: 1 μm).

Contact angle (CA) measurements demonstrated a significant increase from 20° to approximately 55° on both coated sample types (Figure 2a). Similar increases were reported for edible WPI fibril coatings on fruits [25]. Advantageously for cell growth, CA remains lower than 100° [26]. A hydrophilic surface is beneficial for cell adhesion to make sure that proteins from cell culture medium adsorb to the surface in the desired conformation, so that binding sites on the proteins are recognized by the cells. Furthermore, a lower contact angle and higher wettability would facilitate the coating of rough or porous biomaterial surfaces.

SEM images confirmed that fibril coatings withstood washing and drying. Fibrils prepared at pH 3.5 (Figure 2c) appeared to be shorter and less straight than those formed at pH 2 (Figure 2b), in agreement with previous studies [5]. Fibrils were detected by SEM after autoclaving (Supplementary Information, Figure S1); hence, they withstand sterilization. Adhesion of the fibrils to substrates would hinder fibril aggregation and degradation as in previous studies [27,28]; hence, the coating is estimated to be one fibril thick.

**Figure 3:**
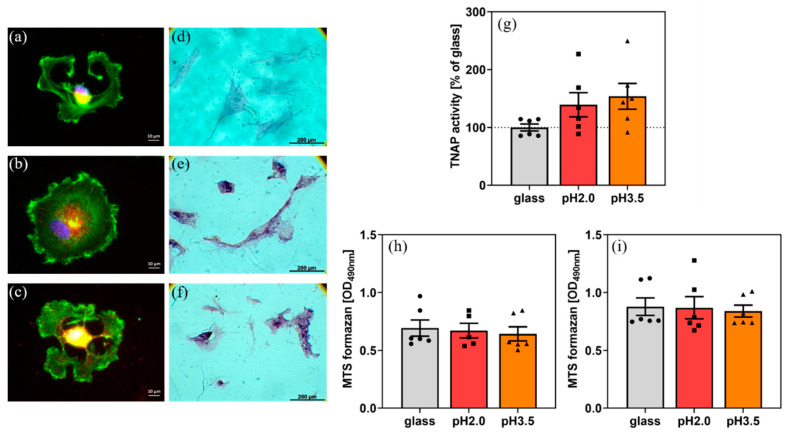
(**a**) Morphology of human bone marrow stromal cells (hBMSC) on (**a**) glass, (**b**) fibrillar coating (pH = 2), (**c**) fibrillar coating (pH = 3.5), 2 h after plating, and (**d**–**f**) TNAP staining at day 11 after plating, respectively. (**g**) TNAP activity on different substrates (day 11) and metabolic activity results at (**h**) day 2 and (**i**) day 4 after plating.

Adhesion of hBMSC was confirmed on the uncoated (Figure 3a) and coated samples (Figure 3b,c). Spreading was superior and tissue non-specific alkaline phosphatase (TNAP) activity (a marker of osteogenic differentiation and linked to the calcium deposition as shown previously [9,29]) was higher on coated samples (Figure 3g). The cells became confluent over the WPI coatings.

hBMSC on coated samples showed 2 h after seeding clear focal adhesion contacts and a well-organized cytoskeleton. A possible reason for that could be that diverse proteins from the serum and cellular in situ-formed proteins adsorbed on the WPI layer and thus promoted initial adhesion. The cell number on coated samples at day 2 and day 4 after seeding (MTS formazan formation is a measure of cell number and can be used as an index of cell proliferation) did not differ substantially from that on glass.

For hBMSC, proper adhesion, spreading and re-organization of the cytoskeleton is an essential prerequisite for proliferation and differentiation of the cells. The fibrillar coating promoted the adhesion and re-organization of the cytoskeleton of hBMSC, did not influence the number of adherent cells, but obviously improved their “quality”, as evidenced by higher TNAP activity.

Hence, we suspect that the coatings induced a superior start of the differentiation program of cells. Analyses concerning the molecular mechanisms are planned for future studies. In the present study, we wished to ascertain whether such a fibrillar coating has advantages for hBMSC adhesion and promotes osteogenic parameters.

Apparently, differences in the yields and morphologies of straight fibrils observed at pH 2 and the worm-like aggregates observed at pH 3.5 do not affect hBMSC. hBMSC metabolic activity increased slightly from day 2 to 4 (Figure 3h,i), but not significantly.

Future work will focus on the preparation of coatings from fibrils obtained at other pH values. It is well known that the typical fibrils with amyloid structure only occur at pH < 3, while worm-like aggregates can be observed at pH 3.5 [6]. Spherical aggregates emerge at pH 4 to 5 [7] and, otherwise, there are also smaller aggregates observed at neutral pH. Besides pH-induced changes, the addition of sodium chloride or the protein concentration can affect the morphology, as well as the addition of solvents [22,30]. The structures have different yields (i.e., portion of amyloid to non-assembled material), but also different processing stabilities. Thus, there is a whole range of conditions that can be used to alter the morphology and to study the correlation between structure and cell behavior in the future.

Another focus of future work will be the extension of the cell biological characterization of the coatings, to elucidate the exact mechanism by which coatings may promote differentiation, and to include cell-induced mineralization. From previous investigations, it is known that an increase in TNAP activity leads to an increase in released phosphate ions into the conditioned medium, and consequently to enhanced mineralization [9], which should be studied in future.

WPI fibrillar coatings can be enhanced by the incorporation of molecules with growth-stimulatory or antimicrobial properties into the coatings, coating thickness and mechanical measurements and substrates more appropriate for bone contact (e.g., titanium alloy). Furthermore, WPI fibrillar coatings should be compared to more commonly used fibrillar coatings of fibronectin and collagen, which are known to promote cell adhesion [14,15,16,17,18,19,20].

## 3. Materials and Methods

WPI (BiPro, Davisco Foods International Inc., Eden Prairie, MN, USA) was dissolved in Milli-Q (2.5 wt%). pH was set to 2 and 3.5 by adding HCl. 15 mL WPI solution was heated (90 °C, 5 h, stirring speed 350 rpm) to induce fibril formation resulting in a fibrillar suspension. Glass substrates (chosen as an inexpensive substrate for this pilot study) were coated with fibrils by adsorption from the suspension. Substrates were rinsed with Milli-Q to remove non-adhered fibrils, air-dried and autoclaved (121 °C, 15 min). SEM, CA measurements and fibrillar yield quantification were performed as described previously [5,7,31,32].

hBMSC was isolated from bone marrow aspirates from donors (males, average 27 ± 5 y) who gave full informed consent (local ethics commission (ethic vote No. EK466112016)), at the Bone Marrow Transplantation Center, University Hospital Dresden, characterized and plated onto samples (5555 hBMSC/cm^2^), as described previously [29,33].

After 2 h, hBMSC morphology was monitored by fluorescence staining of F-actin and phosphorylated focal adhesion kinase, as described previously [8]. Metabolic activity of hBMSC was determined by the standard MTS assay (Cell Titer96 AQueous One Solution Proliferation Assay) (Promega, Mannheim, Germany). Cell culture medium was replaced by fresh medium containing 10% of MTS dye solution. After incubation in a humidified CO_2_ incubator (2 h, 37 °C), 80 µL medium was removed, and absorbance was measured photometrically at 490 nm.

At day 11 after seeding, hBMSC was stained for tissue non-specific alkaline phosphatase (TNAP) enzyme activity with a commercial staining kit (86-R, Sigma). Images were obtained and TNAP enzyme activity was determined, as described previously [8].

Experiments were performed with cells from three different donors (*n* = 3), each in triplicate. Results are presented as mean ± standard error of the mean. Statistical significance was analyzed with GraphPad Prism 8.4 software (Statcon, Witzenhausen, Germany) by ANOVA analysis, with Bonferroni’s post-test.

## 4. Conclusions

Formation of WPI fibrils in solution was strongly pH-dependent; fibrillar yield increased when pH was increased from 2 to 3.5. WPI fibrillar coatings resisted autoclave sterilization and supported the attachment, spreading and differentiation of hBMSC. The pH 2 and pH 3.5 fibrils had an equally positive effect on cell differentiation.

## Supplementary Materials

Supplementary Materials can be found at https://www.mdpi.com/1422-0067/21/15/5544/s1.

## Abbreviations

CA	Contact angle
hBMSC	Human bone marrow stromal cells
SEM	Scanning electron microscopy
TNAP	Tissue non-specific alkaline phosphatase
WPI	Whey protein isolate

## References

[1] Douglas T.E.L., Vandrovcová M., Kročilová N., Keppler J.K., Zárubová J., Skirtach A.G., Bačáková L. (2018). Application of whey protein isolate in bone regeneration: Effects on growth and osteogenic differentiation of bone-forming cells. J. Dairy Sci..

[2] Gupta D., Kocot M., Tryba A.M., Serafim A., Stancu I.C., Jaegermann Z., Pamuła E., Reilly G.C., Douglas T.E.L. (2020). Novel naturally derived whey protein isolate and aragonite biocomposite hydrogels have potential for bone regeneration. Mater. Des..

[3] Keppler J.K., Martin D., Garamus V.M., Berton-Carabin C., Nipoti E., Coenye T., Schwarz K. (2017). Functionality of whey proteins covalently modified by allyl isothiocyanate. Part 1 physicochemical and antibacterial properties of native and modified whey proteins at pH 2 to 7. Food Hydrocoll..

[4] Akkermans C., Venema P., van der Goot A.J., Gruppen H., Bakx E.J., Boom R.M., van der Linden E. (2008). Peptides are building blocks of heat-induced fibrillar protein aggregates of β-lactoglobulin formed at pH 2. Biomacromolecules.

[5] Heyn T.R., Garamus V.M., Neumann H.R., Uttinger M.J., Guckeisen T., Heuer M., Selhuber-Unkel C., Peukert W., Keppler J.K. (2019). Influence of the polydispersity of pH 2 and pH 3.5 beta-lactoglobulin amyloid fibril solutions on analytical methods. Eur. Polym. J..

[6] Keppler J.K., Heyn T.R., Meissner P.M., Schrader K., Schwarz K. (2019). Protein oxidation during temperature-induced amyloid aggregation of beta-lactoglobulin. Food Chem..

[7] Serfert Y., Lamprecht C., Tan C.P., Keppler J.K., Appel E., Rossier-Miranda F.J., Schroen K., Boom R.M., Gorb S., Selhuber-Unkel C. (2014). Characterisation and use of β-lactoglobulin fibrils for microencapsulation of lipophilic ingredients and oxidative stability thereof. J. Food Eng..

[8] Hempel U., Preissler C., Vogel S., Möller S., Hintze V., Becher J., Schnabelrauch M., Rauner M., Hofbauer L.C., Dieter P. (2014). Artificial extracellular matrices with oversulfated glycosaminoglycan derivatives promote the differentiation of osteoblast-precursor cells and premature osteoblasts. BioMed Res. Int..

[9] Hempel U., Matthäus C., Preissler C., Möller S., Hintze V., Dieter P. (2014). Artificial matrices with high-sulfated glycosaminoglycans and collagen are anti-inflammatory and pro-osteogenic for human mesenchymal stromal cells. J. Cell. Biochem..

[10] Knowles T.P.J., Oppenheim T.W., Buell A.K., Chirgadze D.Y., Welland M.E. (2010). Nanostructured films from hierarchical self-assembly of amyloidogenic proteins. Nat. Nanotechnol..

[11] Reynolds N.P., Styan K.E., Easton C.D., Li Y., Waddington L., Lara C., Forsythe J.S., Mezzenga R., Hartley P.G., Muir B.W. (2013). Nanotopographic surfaces with defined surface chemistries from amyloid fibril networks can control cell attachment. Biomacromolecules.

[12] Reynolds N.P., Charnley M., Mezzenga R., Hartley P.G. (2014). Engineered lysozyme amyloid fibril networks support cellular growth and spreading. Biomacromolecules.

[13] Reynolds N.P., Charnley M., Bongiovanni M.N., Hartley P.G., Gras S.L. (2015). Biomimetic topography and chemistry control cell attachment to amyloid fibrils. Biomacromolecules.

[14] Douglas T., Heinemann S., Mietrach C., Hempel U., Bierbaum S., Scharnweber D., Worch H. (2007). Interactions of collagen types I and II with chondroitin sulfates A-C and their effect on osteoblast adhesion. Biomacromolecules.

[15] Geißler U., Hempel U., Wolf C., Scharnweber D., Worch H., Wenzel K.W. (2000). Collagen type I-coating of Ti_6_A_14_V promotes adhesion of osteoblasts. J. Biomed. Mater. Res..

[16] Vandrovcova M., Douglas T.E.L., Heinemann S., Scharnweber D., Dubruel P., Bacakova L. (2015). Collagen-lactoferrin fibrillar coatings enhance osteoblast proliferation and differentiation. J. Biomed. Mater. Res. Part A.

[17] Kim H.-W., Li L.-H., Lee E.-J., Lee S.-H., Kim H.-E. (2005). Fibrillar assembly and stability of collagen coating on titanium for improved osteoblast responses. J. Biomed. Mater. Res. Part A.

[18] Douglas T., Hempel U., Mietrach C., Viola M., Vigetti D., Heinemann S., Bierbaum S., Scharnweber D., Worch H. (2008). Influence of collagen-fibril-based coatings containing decorin and biglycan on osteoblast behavior. J. Biomed. Mater. Res. Part A.

[19] Franke K., Pompe T., Bornhäuser M., Werner C. (2007). Engineered matrix coatings to modulate the adhesion of CD133+ human hematopoietic progenitor cells. Biomaterials.

[20] Gui L., Wojciechowski K., Gildner C.D., Nedelkovska H., Hocking D.C. (2006). Identification of the heparin-binding determinants within fibronectin repeat III1: Role in cell spreading and growth. J. Biol. Chem..

[21] Dziadek M., Douglas T.E.L., Dziadek K., Zagrajczuk B., Serafim A., Stancu I.C., Cholewa-Kowalska K. (2020). Novel whey protein isolate-based highly porous scaffolds modified with therapeutic ion-releasing bioactive glasses. Mater. Lett..

[22] Heyn T.R., Mayer J., Neumann H.R., Selhuber-Unkel C., Kwade A., Schwarz K., Keppler J.K. (2020). The threshold of amyloid aggregation of beta-lactoglobulin: Relevant factor combinations. J. Food Eng..

[23] Pellarin R., Caflisch A. (2006). Interpreting the Aggregation Kinetics of Amyloid Peptides. J. Mol. Biol..

[24] Ye X., Hedenqvist M.S., Langton M., Lendel C. (2018). On the role of peptide hydrolysis for fibrillation kinetics and amyloid fibril morphology. RSC Adv..

[25] Feng Z., Wu G., Liu C., Li D., Jiang B., Zhang X. (2018). Edible coating based on whey protein isolate nanofibrils for antioxidation and inhibition of product browning. Food Hydrocoll..

[26] Bacakova L., Filova E., Parizek M., Ruml T., Svorcik V. (2011). Modulation of cell adhesion, proliferation and differentiation on materials designed for body implants. Biotechnol. Adv..

[27] Gilbert J., Campanella O., Jones O.G. (2014). Electrostatic stabilization of β-lactoglobulin fibrils at increased pH with cationic polymers. Biomacromolecules.

[28] Li C., Born A.K., Schweizer T., Zenobi-Wong M., Cerruti M., Mezzenga R. (2014). Amyloid-hydroxyapatite bone biomimetic composites. Adv. Mater..

[29] Hempel U., Müller K., Preissler C., Noack C., Boxberger S., Dieter P., Bornhäuser M., Wobus M. (2016). Human Bone Marrow Stromal Cells: A Reliable, Challenging Tool for in Vitro Osteogenesis and Bone Tissue Engineering Approaches. Stem Cells Int..

[30] Kayser J.J., Arnold P., Steffen-Heins A., Schwarz K., Keppler J.K. (2020). Functional ethanol-induced fibrils: Influence of solvents and temperature on amyloid-like aggregation of beta-lactoglobulin. J. Food Eng..

[31] Douglas T.E.L., Hempel U., Żydek J., Vladescu A., Pietryga K., Kaeswurm J.A.H., Buchweitz M., Surmenev R.A., Surmeneva M.A., Cotrut C.M. (2018). Pectin coatings on titanium alloy scaffolds produced by additive manufacturing: Promotion of human bone marrow stromal cell proliferation. Mater. Lett..

[32] Li M., Aveyard J., Fleming G., Curran J.M., McBride F., Raval R., D’Sa R.A. (2020). Nitric Oxide Releasing Titanium Surfaces for Antimicrobial Bone-Integrating Orthopedic Implants. ACS Appl. Mater. Interfaces.

[33] Oswald J., Boxberger S., Joergensen B., Bornhaeuser M., Ehninger G., Werner C. (2004). Mesenchymal Stem Cells (MSC) can be differentiated into endothelial cells in vitro. Proceedings of the Transactions—7th World Biomaterials Congress.

