# Supplementary material for: Dairy-Inspired Coatings for Bone Implants from Whey Protein Isolate-Derived Self-Assembled Fibrils
Source: Int J Mol Sci. 2020 Aug 3;21(15):5544. doi: 10.3390/ijms21155544 (PMC7432503; doi:10.3390/ijms21155544)
Supplement: Supplementary file 1 [file ijms-21-05544-s001.pdf]

Supplementary data

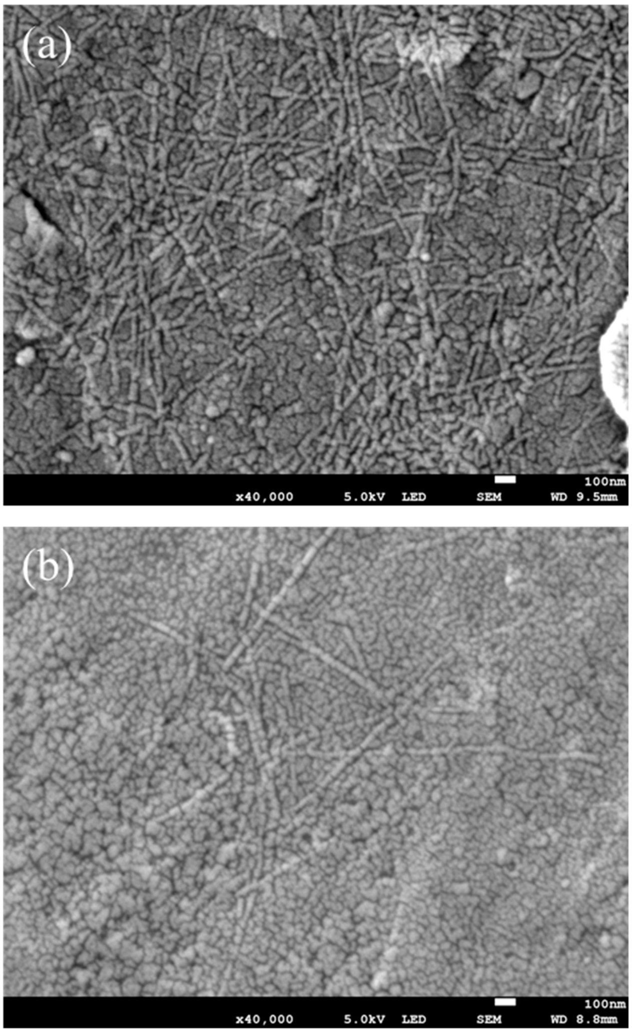

Figure S1. (a) SEM images of fibrillar coatings obtained from WPI solutions at (a) pH 2 and (b) pH 3.5 after autoclaving (magnification: ×40000; scale bar: 100 nm).
